# Effect of changes in the net height, court size, and serve limitations on technical-tactical, physical, and psychological aspects of U-14 female volleyball matches

**DOI:** 10.3389/fpsyg.2023.1341297

**Published:** 2024-01-10

**Authors:** Jose M. Palao, Aurelio Ureña, Maria P. Moreno, Enrique Ortega-Toro

**Affiliations:** ^1^Department of Health, Kinesiology and Sport Management, University of Wisconsin-Parkside, Kenosha, WI, United States; ^2^Sports Performance Analysis Association, SPAA, Faculty of Sports Sciences, University of Murcia, Murcia, Spain; ^3^Department of Physical and Sports Education, Faculty of Sports Sciences, University of Granada, Granada, Spain; ^4^Faculty of Sport Science, Regional Campus of International Excellence “Campus Mare Nostrum”, University of Murcia, Murcia, Spain

**Keywords:** team sport, youth, constraints, scaling, development, competition rules

## Abstract

**Introduction:**

The objective was to analyze the effect of a reduction of the net height and the court size and serve limitations on the technical-tactical actions, physical actions, and psychological aspects in youth volleyball players.

**Methods:**

The sample was 29 under-14 female volleyball players (three regional club teams). A quasi-experimental design was implemented to assess the effect of modification in three tournaments. The independent variables were: a) official rules tournament (no changes in the rules), b) Experimental Tournament 1 (reduction in the net height from 2.10 m to 2 m, no jump serves, and a maximum of two serves per player and rotation), and c) Experimental Tournament 2 (reduction in net height from 2.10 m to 2 m, reduction in court size from 9 × 9 m to 8 × 8 m, no jump serves, and a maximum of two serves per player and rotation). The dependent variables were: ball contact done (type), quality and efficacy of the technical actions, team game phases occurrence, quality and efficacy, continuity index, number of jumps, player's jump load in the take-off and landing, number of hits, average heart rate, Rate of Perceived Effort, time between ball contacts, serve velocity, perceived individual and collective self-efficacy, perceived enjoyment, and perceived satisfaction.

**Results:**

Experimental Tournament 1 involved an increase in the efficacy of serves and a decrease in the efficacy of side-out phases. The imbalance between serve and reception did not impact game continuity but reduced the attack and blocks. Experimental Tournament 2 involved a decrease in the efficacy of serves and an increase in the efficacy of side-out phases. The balance between serve and reception increased reception efficacy, the occurrence of attacks and blocks, game continuity, and players' effort. players' effort.

**Discussion:**

Scaling the net and court and adapting the serve rules (Experimental Tournament 2) resulted in game dynamics for these U-12 teams that were more similar to those of posterior stages of player through the balance between serve and reception and the adaptation of the net height and court size.

## Introduction

Volleyball is a net sport and team sport in which players cannot catch the ball. This characteristic makes the learning process difficult due to the requirement of physical and motor skills that players must have to volley the ball. Rules allow teams to make three contacts with the ball. Teams use these three contacts to carry out a cyclical sequence of actions to control and send the ball to the opponent's court with the intent of obtaining a point by making the ball contact the opponent's court. The first action of a rally is the serve which sends the ball to the opponent's court (reception). The first team's contact attempts to control the ball sent by the opponent. The second contact attempts to create the best conditions to send the ball to the opponent's court (set). Finally, the third contact tries to put the ball in contact with the opponent's court (attack). The other team attempts to neutralize the opponent's attack near the net by making a block, which does not count in the cycle of three contacts allowed per team. After that, the team will try to neutralize the attack (defense) and start the cycle again, building their offense. The play continues with this cycle until one of the teams gets a point. Through the developmental process, the goal of training and competition is to provide players with appropriate experiences for their level of maturity and skill to achieve the purpose of the game. Competition rules for each age group establish the reference conditions that players will have in their games. The manipulation of the game rules throughout the players' development seeks to increase children's participation through actions that are appropriate to their learning and physical, technical, and psychological characteristics (Bunker and Thorpe, 1982; Kirk and MacPhail, 2002; Buszard et al., 2016). In volleyball, federations or school organizations establish the competition formats and rules adapted to the players' development. In the 1960s, an adaptation of volleyball was developed for younger age groups, called mini volley (Baacke, 1975). This adapted sport involves scaling the structural aspect of the game, like the number of players, net height, ball size, and court size (Buszard et al., 2016). For older players (U-12 and older), volleyball is played with similar rules to adults, except that the net height progresses until the adult height is reached by U-16 players. The evolution of the net height and other rule adaptations, such as the use of the libero (player who specializes in defense) and serve limitations (e.g., no jump serve allowed), change for each country or region. Currently, there is no evidence of the adequacy and impact of these rules for each age group. The influence of these rules on the development of the youth player is unknown.

There are studies that show the effect of the manipulation of different constraints (Newell, 1986) on the game during training: type of ball, court size, number of players, ball retention, etc. (i.e., Castro et al., 2022; Halouani et al., 2023). These studies had the goal of providing information to physical education teachers and volleyball coaches about the manipulation of different task constraints. For example, the manipulation of the net height affects the trajectory of the ball which impacts the time that players have to intercept the ball (Palao and Guzman, 2008). The net height should be established according to the anthropometric and physical characteristics of players. An increase in the net height increases the ball parabola and provides players with more time to the intercept ball. This, in theory, improves the players' chances of intercepting the movement, the quality of the reception and defense actions, and the continuity of the game. However, the actions of serve and spike, in which players try to put the ball in contact with the court, are affected. A higher net changes the trajectory of the serve and attack, and it can impact the speed of these movements. The manipulation of the court size affects the area that players have to cover and their ability to intercept the ball. An increase in the court size made it more difficult for players to intercept the ball and decreased the quality of the first team's ball contact. The reduction of the court area limits the ball trajectories, reduces ball speed, and increases the chances of intercepting the ball (Ronglan and Grydeland, 2006; Rodrigues et al., 2022). The specific effect of court manipulation changes for each court size (Rocha et al., 2020a,b; Rodrigues et al., 2022). The changes of the court size are influenced by the number of players: m^2^ per player (Halouani et al., 2023). The interaction of the manipulation of net height and court size on small-side games results in different changes in the game's continuity, quality, and efficacy of the technical-tactical actions. These manipulations could change the movements, physical actions, and other aspects that are impacted by the way players execute their actions, such as motivation or self-efficacy. An increase in the game's continuity and the efficacy of the terminal actions (spike and block) increase the motivation of volleyball players (García-de-Alcaraz et al., 2022). In the review carried out, no evidence was found regarding the impact of the serve and spike with different net heights and court changes for younger players in competition. The studies that were found analyzed the effect of net height and court size manipulation on small-sided games.

This study tries to provide evidence regarding the possibility of adapting the rules to the players' needs during the development process. The low number of experimental studies about the progression of the game rules' adaptation makes it difficult to know if the current competition format for each age group is the most adequate option or not. The information currently available in other sports shows that a progressive adaptation of the normative competition could result in qualitative and quantitative improvements of the realization of the technical-tactical actions in competition (i.e., Lapresa et al., 2010; García-Angulo et al., 2020a,b; Gimenez-Egido et al., 2020; Ortega-Toro et al., 2021). Knowing the impact of the manipulation of the net height and court size could contribute to the development of competition rules that allow for better development of youth volleyball players according to their maturity and skills. Knowledge of the effect of these rule changes will allow various stakeholders to consider the possibility of developing different proposals for competition rules for youth volleyball players. Using the evolution of the players through their developmental process as a reference (i.e., García-de-Alcaraz et al., 2017; Echeverria et al., 2019), the experimental hypothesis is that scaling the net and court and adapting the serve rules would increase the efficacy and quality of the technical and tactical actions and players' participation in the game. The objective of this study was to analyze the effect of a reduction of the net height (from 2.10 to 2 m) and the court size (from 9 × 9 m to 8 × 8 m) and serve limitations on the technical-tactical actions, physical actions, and psychological aspects in youth volleyball players.

## Materials and methods

### Participants

The sample was 29 under-14 female volleyball players belonging to three amateur teams from the U-14 age group (regional club competition). The characteristics of the players were the following: average age of 13.4 ± 0.68 years; average height of 1.63 ± 0.96 m; average weight of 55.5 ± 7.9 kg; 2.85 ± 0.31 of average training sessions per week; 1.37 ± 0.44 of average hours of session time; and 3.21 ± 0.85 years of experience. All players in the study had reached puberty by the moment of participation in the study. Guardians of the players were informed of the study and provided written consent. Players played three tournaments using three competition formats (official rules, modified rules option #1, and modified rules option #2). A total of 5,315 ball actions done by the players in nine matches were analyzed. The study was approved by the University Ethics Committee of the research group that carried out the study (ID 1944/2018).

### Design and variables

A quasi-experimental design was implemented to assess the effect of modification in: the height of the net (2.10–2.00 m), type of serve (no jump serves were allowed), limitations on total consecutive serves by players (maximum of two serves per player and rotation), and court size (9 × 9 m to 8 × 8 m). The independent variable was the game format (rules). There were three levels: official rules (Control Tournament) and modified rules (Tournaments #1 and #2). The differences between the official and modified rules were the following: net height (2.10 m vs. 2.00 m), size of the field (9 × 9 m vs. 8 × 8 m), and limitations on the serves done by players (unlimited vs. maximum of two consecutive serves in each rotation) and type of serve (jump serves were not allowed). When the limit of serves was reached, the serving team rotated. Table 1 shows the rules that were used in the different competition formats. The first tournament was played according to the official state rules for the U-14 competitions established by the Spanish National Federation. In the second and third tournaments, a modification of the official U-14 volleyball rules was implemented. The players studied played the tournaments with their usual teams.

**Table 1:** Description of the rules implemented in the tournaments (official rules and experimental groups).

**Rules**	**Official rules**	**Modified rules Tournament 1**	**Modified rules Tournament 2**
Number of players	6 players (no libero allowed)	6 players (no libero allowed)	6 players (no libero allowed)
Net height (m)	2.10 m	2.00 m	2.00 m
Field size (m)	9 × 9 m	9 × 9 m	8 × 8 m
Ratio of m^2^ per player	13.5 m^2^	13.5 m^2^	10.6 m^2^
Serve (*n*)	No limitations number serves	Max two serves per player after that team's rotation	Max two serves per player after that team's rotation
Serve (type)	No limitations	No allowed jump serve	No allowed jump serve
Ball size (m)	0.66 m	0.66 m	0.66 m
Format	Best of 5 sets	Best of 5 sets	Best of 5 sets
Points	25 points (5th set, 15 points)	25 points (5th set, 15 points)	25 points (5th set, 15 points)
Score system	Rally score point system	Rally score point system	Rally score point system

The dependent variables were:

a) Ball contacts done (occurrence of serves, receptions, sets, attacks, blocks, and defenses);
b) Quality of the technical actions (scale 0–5). The quality of the technical actions was assessed according to the way of execution of the players' actions (Table 2). Each ball action had a quality score according to the aspects of the execution that were done properly (scale 0–5, where 0 points involved that no aspects were done properly and 5 points that all aspects were done properly);
c) Efficacy of the technical actions (scale 0 to 3–4). The efficacy of the technical actions was established according to the effect of the action on the rally (scale 0 to 3–4), in which 0 was an error, 1–3 was a continuity that allows the opponents or the own team to play the ball (not limit attack, limit attack, and does not allow to attack, 1, 2, and 3 respectively) and 4 was a point. For reception, set, and defense, a scale of 0–3 was used because these actions do not allow to get a point (continuity or preparation actions). For serve, attack, and block (terminal actions), a scale of 0 to 4 was used. The efficacy scale was developed by Coleman et al. (1969) and adapted and validated by Palao et al. (2015);
d) Coefficient efficacy (coefficient −1 to 1). A coefficient efficacy was calculated with the efficacy of each variable using the formula: occurrence of the highest efficacy minus the occurrence of the worst efficacy divided by the occurrence of the action;
e) Team game phases (serve phase, phases done by the team in reception-set-attack, and phases done by the team in defense-set-counter attack phase);
f) Team game phases efficacy (scale 0–4). The efficacy of the technical actions was established according to the effect of the action on the rally (scale 0–4), in which 0 was an error, 1–3 was a continuity that allows the opponents or the own team to play the ball (not limit attack, limit attack, and does not allow to attack, 1, 2, and 3 respectively) and 4 was a point;
g) Team game phases quality (scale 0–6). The quality of the team game phases was assessed according to players' position in the court in the phase. Each phase had a quality score according to the players that were in the position established in the phases of reception, in the initial position of defense and in the final defense position (scale 0 to, where 0 points involved that no players were in the proper position and 6 points that all players were in their established positions);
h) Continuity index (occurrence of rallies that involved continuity and percentage). The continuity index was established using the rallies per play (the number of times that the ball passed over the net);
i) Number of jumps (occurrence);
j) Player's jump load in the take-off and landing (G). The player jump load was assessed with a 3D gyroscope that players wore on their backs (WIMU, Hudl, Chicago, USA);
k) Number of hits (occurrence);
l) Average heart rate in each rally (beats per minute). The heart rate for each player was measured using a chest band (Garmin band, Olathe, Kansas, USA). The average heart rate was collected at the end of each rally;
m) Rate of Perceived Effort (scale 1–10). The rate of Perceived Exertion was collected at the end of each match using a color pictorial scale of 1–10 (adapted from Groslambert et al., 2001);
n) Time between ball contacts (seconds);
o) Serve velocity (km/h). The serve speed was calculated using two radar devices (Pocket Radar, Santa Rosa, California, USA) located behind the serve zones (peak speed);
p) Perceived individual and collective self-efficacy (scale 0–140). The individual-specific self-efficacy and collective self-efficacy were assessed using the “Questionnaire of specific self-efficacy and collective self-efficacy in volleyball” (adapted from Ryckman et al., 1982; Godoy, 1992). The “Questionnaire of Specific Self-efficacy and Collective Self-Efficacy in Volleyball” had 32 closed questions (10-item Likert scale) related to individual and collective self-efficacy (16 questions, respectively). Self-efficacy was assessed at the end of each match;
q) Perceived enjoyment (scale 1–10). Perception of players' experience was assessed after each tournament. Players were asked whether they had experienced a higher, equal, or lower self-efficacy than in the previous tournament in the different actions of the game; and
r) Perceived satisfaction (scale 1–10); the satisfaction was assessed using a scale of 0–10 at the end of each match.

**Table 2:** Description of criteria used to evaluate the quality of the technical actions (adapted from Palao and Hernández, 2010).

**Action**	**Description (scale 0–5)**
Serve	1. The height and trajectory of the ball tossed do not limit serve execution 2. Lower body participation of the kinetic chain of the execution (fluid, coordinative, and sequence movements) 3. Upper body participation of the kinetic chain of the execution (fluid, coordinative, and sequence movements) 4. Way in which the ball is contacted (part of the hand, the height of contact, and place of contact related to the shoulder) 5. Follow-up movement (transfer energy and incorporate into the game)
Reception & defense	1. Win the ball (the player is behind the ball before the contact because s/he perceived and intercepted the ball trajectory) 2. Orientation to the destination pass zone 3. Contact surface (first third of the forearm with arms extended, together and the same height) 4. Contact height (umbilicus height) 5. Kinetic chain (whole body participation to accelerate the ball to the destination zone)
Set	1. Win the ball (the player is behind the ball before the contact because s/he perceived and intercepted the ball trajectory) 2. Orientation to the destination pass zone 3. Contact surface (index and thumb of both hands form a triangle. Ball is contacted with the fingers and not with the palm) 4. Contact height (ball is contacted above of the line of eyes) 5. Kinetic chain (whole body participation with or without jumping to accelerate the ball to the destination zone)
Attack	1. Running approach (the approach allows player to intercept the ball without affecting her/his posterior actions) 2. Jump kinetic chain (realization of the pre-jump, body position, and sequence of the take-off) 3. Hit kinetic chain (Swing and cocking of the arm) 4. Ball contact (part of the hand, height of contact, and place of contact related to the shoulder) 5. Follow-up movement (follow-up movement of the arm swing, balance landing with two leg and arm swing in the landing)
Block	1. Appreciate and intercept ball trajectory (temporally and spatially displacement and location regarding the attack) 2. Jump kinetic chain (realization of the approach (if necessary), body position, and sequence of the take-off) 3. Arms actions (progressive action of sealing the net with the arms) 4. Ball contact and hands orientation (Both hands open, rigid, and orientated to the court) 5. Follow-up movement (follow-up movement of the arm swing and balance landing with two legs)

### Procedure

The design and validation of the “Questionnaire of specific self-efficacy and collective effectiveness in volleyball” (Supplementary Appendix 1) were done adapting a basketball survey designed and validated by García-Angulo et al. (2020a,b). The design and validation followed the Delphi method (panel of experts, college professors in Sport psychology) and an experts' evaluation. In the first phase, a panel of five expert judges participated. Two rounds were done between the panel of experts. The analysis done by the experts was qualitative. The second phase involved doing an expert validation through four experts (Ph.D. in Kinesiology and experience in coaching volleyball) who evaluated the accuracy, precision, and wording of each section of the measuring instrument. The V of Aiken was used to calculate the content validity, obtaining minimum values of 0.95. For establishing the reliability, Test-Retest reliability was assessed with a pilot study using the test-retest technique. The pilot study was done with 21 subjects, with similar characteristics to the sample. The minimum values obtained from the intraclass correlation coefficient were 0.96 (Weir, 2005).

The data was recorded in three tournaments. The tournaments were played after the end of the official regular season. All tournaments were played in an indoor pavilion and in similar atmospheric conditions. In total, nine matches were played in the three tournaments, three matches in each tournament (two matches per team and tournament). The competition system was round robin. The order of the confrontations was the same in the different tournaments. Team followed their match routines and warm-up. The actions developed by the players were recorded with two fixed digital cameras (50 fps) from an elevated rearview (lateral and posterior). The actions were recorded and analyzed by one trained observer (Master in Sports Science with at least 2-years of experience in match analysis and volleyball). The observer was trained with the observation instrument. After the training period, inter- and intra-observer reliability were calculated (Cohen's Kappa for the nominal variables and Inter-class correlation coefficient and Pearson correlation for the continues variables). To calculate the intra-observer reliability, another researcher was used as a reference. The researcher held a sports science degree and had more than 10 years of experience in sports analytics. The reliability of the observers was measured before and after the observation. For the nominal variables, the lowest level of interobserver reliability was 0.84, and the lowest level of intra-observer reliability was 0.93 (Cohen's Kappa). For the continuous variables, the lowest level of inter-observer and intra-observer reliability was 0.96 (Inter-class correlation coefficient and Pearson correlation). Players wore an accelerometer (WIMU, Hudl, Chicago, USA) and heart rate chest band to monitor the jump load and their heart rate (Garmin band, Olathe, Kansas, USA). The radar devices were located in the middle of the baselines to record the peak speed of each serve (Pocket Radar, Santa Rosa, California, USA). Rate of Perceived effort was registered at the end of each match. Players' self-efficacy was measured at the end of each tournament (15–30 min after the last game of each tournament). In all the tournaments the same procedures were used, and the questionnaires were explained and provided by the same researcher.

### Data analysis

Descriptive (means, standard deviation and percentages) and inferential statistics of the data were calculated. Data of the quality of the team game phases were expressed in the results in percentages. Data of the different self-efficacy assessed (specific self-efficacy, and collective self-efficacy) are presented in the results section on a scale 0 to 100 in order to allow the comparison of the impact of each experimental condition on self-efficacy. To assess the normality of data of the continuous and categorical variables, Kolmogorov Smirnov test and Chi square test were used, respectively. Data assumes no normal distribution which led to the use of non-parametric test. To measure the difference between the different tournaments in continuous variables, Wilcoxon test or U Mann-Whitney test were used. To measure the magnitude of the effect size, the Rank Biserial Correlation (RBC) was used, using the following classification (Coolican, 2017): minimal effect (RBC < 0.10), moderate effect (0.10 < RBC < 0.30) and strong effect (RBC > 0.50). Rank Biserial Correlation, measures the magnitude of the effect size for comparative studies, as rank correlation, when using the Wilcoxon rank test (Dominguez-Lara et al., 2019). To measure the difference between the different tournaments in categorical variables, Pearson Chi-square test was used. The effect size of these differences was established using the V of Cramer. The level of significance was set at *p* < 0.05. To measure the magnitude of the effect size the eta square (η^2^) was used using the following classification (Ferguson, 2009): no effect (η^2^ < 0.04), minimum effect (0.04 < η^2^ < 0.25), moderate effect (0.25 < η^2^ < 0.64) and strong effect (η^2^ > 0.64). The statistical analysis was completed with JAMOVI statistics software (version 2.4.8).

## Results

Regarding the impact of the experimental rules on the actions taken by players, each tournament resulted in specific changes in the occurrence, quality, and efficacy of the actions (Table 3). In the Control Tournament, 21.5% of the serves were done using the jump serve technique and in 58 out of 251 serves (23%) were done by players after serving more than two serves per rotation. In experimental tournaments 1 and 2, the limit of serves was reached in 10–11 out 86–88 possible situations (≅11%−12%, respectively). In Tournament 1, there were significantly fewer attacks than in Tournament 2 (*p* < 0.01, ES = 0.688), significantly fewer blocks than in both the Control Tournament (*p* < 0.01, ES = 0.696) and Tournament 2 (*p* < 0.001, ES = 0.969), significantly higher quality of the defense actions than in both the Control Tournament (*p* < 0.09, ES = 0.389) and Tournament 2 (*p* < 0.05, ES = 0.442), significantly lower block efficacy than in the Control Tournament (*p* < 0.01, ES = 0.696), a significantly higher efficacy coefficient for the serve than in Tournament 2 (*p* < 0.01, ES = 0.652), and significantly higher efficacy of the defense actions than in both the Control Tournament (*p* < 0.05, ES = 0.535) and Tournament 2 (*p* < 0.05, ES = 0.521). These differences had a medium-large effect size. In Tournament 2, there were significantly more sets than in Tournament 1 (*p* < 0.01, ES = 0.486), significantly more attacks than in both the Control Tournament (*p* < 0.01, ES = 0.588) and Tournament 1 (*p* < 0.01, ES = 0.688), significantly more blocks than in both the Control tournament (*p* < 0.001, ES = 0.920) and Tournament 1 (*p* < 0.001, ES = 0.969), significantly more defense than in Tournament 1 (*p* < 0.01, ES = 0.519), a significantly higher quality of serve execution than in the Control Tournament (*p* < 0.05, ES = 0.588), a significantly higher quality of reception execution than in both the Control Tournament (*p* < 0.01, ES = 0.498) and Tournament 1 (*p* < 0.01, ES = 0.637), a significantly lower serve efficacy than in Tournament 1 (*p* < 0.01, ES = 0.585), a significantly lower coefficient efficacy for serve than in both the Control tournament (*p* < 0.05, ES = 0.535) and Tournament 1 (*p* < 0.01, ES = 0.652), a significantly higher coefficient efficacy for reception than in the Control Tournament (*p* < 0.01, ES = 0.626), and a significantly coefficient efficacy for defense than in the Control Tournament (*p* < 0.01, ES = 0.521). These differences had a medium-large effect size.

**Table 3:** Effect of rules changed on execution of the players' ball contact.

	**Control Tournament (TC) Net 2.10 m/court 9** × **9 m**		**Tournament 1 (T1) Net 2.00 m/court 9** × **9 m**		**Tournament 2 (T2) Net 2.00 m/court 8** × **8 m**		**ES (RBC)**		
**Variables**	***X***	**SD**	***X***	**SD**	***X***	**SD**	**TC-T1**	**TC-T2**	**T1-T2**
**Occurrence (** ***n*** **)**									
Serve	9.30	5.86	9.56	5.00	10.00	5.28	n.s.	n.s.	n.s.
Reception	7.59	7.44	7.37	6.51	8.19	7.85	n.s.	n.s.	n.s.
Set	10.07	14.98	9.66	18.49	12.68	23.65	n.s.	n.s.	0.486^*^
Attack	10.07	8.26	9.52	7.52	13.22	11.34	n.s.	0.588^**^	0.688^**^
Block	9.15	10.55	6.67	7.73	15.56	14.70	0.696^**^	0.920^***^	0.969^***^
Defense	10.67	5.40	9.78	6.98	13.26	9.78	n.s.	n.s.	0.519^**^
**Quality of execution (scale 0–5)**									
Serve	3.77	0.70	3.93	0.60	4.01	0.57	n.s.	0.588^*^	n.s.
Reception	1.92	0.83	1.74	0.81	2.39	0.89	n.s.	0.498^*^	0.637^**^
Set	3.02	0.45	3.21	0.54	3.06	0.48	n.s.	n.s.	n.s.
Attack	2.34	0.85	2.34	0.82	2.17	0.96	n.s.	n.s.	n.s.
Block	2.06	0.36	2.24	0.60	2.21	0.51	n.s.	n.s.	n.s.
Defense	1.68	0.52	1.90	0.63	1.56	0.70	0.389^#^	n.s.	0.442^*^
**Efficacy (scale 0 to 3–4)** ^a^									
Serve	1.97	0.42	2.41	1.16	1.74	0.50	n.s.	0.585^*^	n.s.
Reception	1.46	0.55	1.40	0.51	1.64	0.49	n.s.	n.s.	n.s.
Set	1.90	0.41	1.92	0.65	1.97	0.40	n.s.	n.s.	n.s.
Attack	1.98	0.63	2.06	0.73	1.90	0.59	n.s.	n.s.	n.s.
Block	1.94	0.81	1.60	0.67	1.72	0.45	0.459^#^	n.s.	n.s.
Defense	1.42	0.38	1.54	0.35	1.36	0.51	n.s.	n.s.	n.s.
**Coefficient efficacy (scale** −**1 to 1)**									
Serve	0.01	0.23	0.01	0.32	−0.09	0.23	n.s.	0.535^*^	0.652^**^
Reception	−0.25	0.27	−0.13	0.27	−0.03	0.24	n.s.	0.626^**^	n.s.
Set	0.15	0.17	0.21	0.40	0.13	0.22	n.s.	n.s.	n.s.
Attack	0.07	0.27	0.09	0.35	−0.02	0.29	n.s.	n.s.	n.s.
Block	0.06	0.17	−0.01	0.32	−0.04	0.09	n.s.	n.s.	n.s.
Defense	−0.17	0.24	−0.04	0.17	−0.20	0.31	0.535^*^	n.s.	0.521^*^

^***^p-value < 0.001.

^**^p-value < 0.01.

^*^p-value < 0.05.

^#^*p*-value < 0.09.

n.s., no significant; ES (RBC), effect size (Rank Biserial Correlation); TC-T1, Control Tournament vs. Tournament 1; TC-T2, Control Tournament vs. Tournament 1; T1-T2, Tournament 1 vs. Tournament 2.

^a^Scale 0–3 for the continuity actions (reception, set and defense) and scale 0–4 for the terminal actions (serve, attack and block).

Regarding the impact of the experimental rules on the tactical team actions (Table 4), in Tournament 1, there were significantly lower efficacy of the side-out than in Tournament 2 (*p* < 0.05, ES = 0.066) and significantly higher quality of the initial defense position than Control Tournament (*p* < 0.01, ES = 0.128). These differences had a minimum effect size. In Tournament 2, there were significantly lower efficacy of the serve phase than in both the Control Tournament (*p* < 0.05, ES = 0.091) and Tournament 1 (*p* < 0.01, ES = 0.097), a significantly higher efficacy of the side-out than in both the Control Tournament (*p* < 0.05, ES = 0.066) and Tournament 1 (*p* < 0.001, ES = 0.109), a significantly higher quality of the initial defense position than Control Tournament (*p* < 0.01, ES = 0.077), and a significantly higher continuity than Control Tournament (*p* < 0.001). These differences had a minimum effect size.

**Table 4:** Effect of rules changed on tactical team actions.

	**Control Tournament (TC) Net 2.10 m/court 9** × **9 m**		**Tournament 1 (T1) Net 2.00 m/court 9** × **9 m**		**Tournament 2 (T2) Net 2.00 m/court 8** × **8 m**		**ES (RBC)**		
**Variables**	***X***	**SD**	***X***	**SD**	***X***	**SD**	**TC-T1**	**TC-T2**	**T1-T2**
**Efficacy team game phases (scale 0–4)**									
Serve	2.06	1.28	2.06	1.27	1.85	1.15	n.s.	0.091^*^	0.097^*^
Reception-set-attack	1.80	1.06	1.72	1.05	1.92	1.03	n.s.	0.066^*^	0.109^***^
Defense & counterattack	1.78	1.05	1.91	1.09	1.83	1.07	n.s.	n.s.	n.s.
**Quality of execution team game phases (%)**									
Reception-set-attack	96.28	8.91	96.16	8.76	96.31	8.16	n.s.	n.s.	n.s.
Initial defense position	77.01	21.46	82.02	18.97	80.74	17.21	0.128^**^	0.077^**^	n.s.
Final defense position	87.87	17.41	89.38	17.32	90.14	16.43	n.s.	n.s.	n.s.
Continuity (*n*)	*N* = 1,288	79.46%	*N* = 1,266	79.62%	*N* = 1,778	84.55%	*p* < 0.001	T2 < (TC = T1)	

^***^p-value < 0.001.

^**^p-value < 0.01.

^*^p-value < 0.05.

n.s., no significant; ES (RBC), effect size (Rank Biserial Correlation); TC-T1, Control Tournament vs. Tournament 1; TC-T2, Control Tournament vs. Tournament 1; T1-T2, Tournament 1 vs. Tournament 2.

Regarding the impact of the experimental rules on players' physical actions (Table 5), in Tournament 1, there were significantly fewer jumps than in both the Control Tournament (*p* < 0.05, ES = 0.518) and Tournament 2 (*p* < 0.001, ES = 0.958), significantly fewer hits than in Tournament 2 (*p* < 0.05, ES = 0.552), significantly higher heart rate than in both the Control Tournament (*p* < 0.001, ES = 0.134) and Tournament 2 (*p* < 0.001, ES = 0.085), significantly less time for reception than in the Control Tournament (*p* < 0.05, ES = 0.464), and significantly more time for block than in the Control Tournament (*p* < 0.05, ES = 0.481). These differences had a medium-large effect size, except for heart rate that had minimum effect size. In Tournament 2, there were significantly higher jumps than in both the Control Tournament (*p* < 0.001, ES = 0.763) and Tournament 1 (*p* < 0.001, ES = 0.958), significantly higher hits than in both the Control Tournament (*p* < 0.01, ES = 0.584) and Tournament 1 (*p* < 0.05, ES = 0.552), significantly higher heart rate than the Control Tournament (*p* < 0.001, ES = 0.055), significantly lower heart rate than Tournament 1 (*p* < 0.001, ES = 0.085), significantly less time between serve and reception than in both the Control Tournament (*p* < 0.05, ES = 0.498), significantly less time for between set and attack actions than in both the Control Tournament (*p* < 0.05, ES = 0.521) and Tournament 1 (*p* < 0.05, ES = 0.474), significantly less time for between attack and block actions than in both the Control Tournament (*p* < 0.01, ES = 0.681), and significantly lower serve speed than in the Control Tournament (*p* < 0.001, ES = 0.107). These differences had a medium-large effect size, except for heart rate that had minimum effect size. Regarding the impact of the experimental rules on the psychological aspects (Table 6), no significant differences were found between the tournament control and the experimental tournaments.

**Table 5:** Effect of rules changed on physical actions done by players.

	**Control Tournament (TC) Net 2.10 m/court 9** × **9 m**		**Tournament 1 (T1) Net 2.00 m/court 9** × **9 m**		**Tournament 2 (T2) Net 2.00 m/court 8** × **8 m**		**ES (RBC)**		
**Variables**	***X***	**SD**	***X***	**SD**	***X***	**SD**	**TC-T1**	**TC-T2**	**T1-T2**
Jumps (*n*)	16.7	15.6	11.7	10.5	22.1	18.5	0.518^*^	0.763^***^	0.958^***^
Jump load (g)	3.68	0.49	3.85	0.37	3.67	0.42	n.s.	n.s.	n.s.
Landing load (g)	5.23	0.84	5.08	1.11	5.00	1.05	n.s.	n.s.	n.s.
Hits (*n*)	19.37	12.2	18.10	11.36	22.39	15.25	n.s.	0.584^**^	0.552^*^
Heart rate (bpm)	155	19.9	162	20.4	159	16.0	0.134^***^	0.055^***^	0.085^***^
RPE (scale 1–10)	3.21	1.57	3.31	1.81	3.77	1.58	n.s.	n.s.	n.s.
**Temporality (s)**									
Reception	1.32	0.13	1.27	0.10	1.29	0.09	0.464^*^	0.498^*^	n.s.
Set	1.15	0.10	1.52	0.23	1.48	0.15	n.s.	n.s.	n.s.
Attack	1.44	0.10	1.42	0.12	1.40	0.10	n.s.	0.521^*^	0.474^*^
Block	2.20	0.20	2.07	0.20	1.95	0.27	0.481^*^	0.681^**^	n.s.
Defense	1.07	0.09	1.13	0.20	1.06	0.17	n.s.	n.s.	n.s.
Serve speed (km/h)	51.4	6.52	50.2	6.53	49.5	6.20	n.s.	0.107^***^	n.s.

^***^p-value < 0.001.

^**^p-value < 0.01.

^*^p-value < 0.05.

n.s., no significant; ES (RBC), effect size (Rank Biserial Correlation); TC-T1, Control Tournament vs. Tournament 1; TC-T2, Control Tournament vs. Tournament 1; T1-T2, Tournament 1 vs. Tournament 2.

**Table 6:** Effect of rules changed on psychological aspects.

	**Control Tournament (TC) Net 2.10 m/court 9** × **9 m**		**Tournament 1 (T1) Net 2.00 m/court 9** × **9 m**		**Tournament 2 (T2) Net 2.00 m/court 8** × **8 m**		**ES (RBC)**		
**Variables**	***X***	**SD**	***X***	**SD**	***X***	**SD**	**TC-T1**	**TC-T2**	**T1-T2**
Individual self-efficacy (%)	71.85	12.86	71.22	10.02	71.97	10.34	n.s.	n.s.	n.s.
Collective self-efficacy (%)	77.19	11.20	77.24	13.24	77.37	11.58	n.s.	n.s.	n.s.
Enjoyment (scale 1–10)	8.41	2.03	8.71	1.88	8.60	1.38	n.s.	n.s.	n.s.
Satisfaction (scale 1–10)	6.79	1.73	7.11	2.50	7.00	1.73	n.s.	n.s.	n.s.

n.s., no significant; ES (RBC), effect size (Rank Biserial Correlation); TC-T1, Control Tournament vs. Tournament 1; TC-T2, Control Tournament vs. Tournament 1; T1-T2, Tournament 1 vs. Tournament 2.

## Discussion

The objective of this study was to analyze the effect of a reduction of the net height, limitations on the serve, and a reduced playing space on the technical, tactical, as well as physical actions and psychological aspects in youth female volleyball matches. Two experimental tournaments were carried out to test the implications of these rule changes on U-14 female players. Each rule change involved a different effect on the game. The reduction of the net height without changing the court size facilitated the serve action, as players increased the efficacy of their serves. Although serve speed did not increase, players in reception had less time to intercept the ball from the serve. The reduction in the reception efficacy affected the way that the offense was built and reduced the number of attacks, the side-out efficacy, and the blocks were done. The speed of the game increased. Blockers had less time to block, there were fewer blocks, and their efficacy decreased. As a result of the less-efficient attack, there was an increase in the quality of the execution and efficacy of the defense actions. The change in the game dynamics involved an increase in the players' heart rate and a reduction in the number of the players' jumps and hits. The increase in the speed of the game and in the defense participation involved players adopting better defense positions before the opponent's attack was executed (defense tactical system). The changes in the net height did not involve changes in the g-force in the take-off or the landing of the attack. The balance between serve and reception is critical to develop the offense. Throughout players' development, there is an improvement in the ability of the receivers to neutralize the serve, which improves the ability to build the side-out (García-de-Alcaraz et al., 2017; Echeverria et al., 2019). This impacts game dynamics because it increases the continuity of the game (i.e., times that the ball passes over the net). The changes observed in experimental tournament 1 show the opposite tendency. The efficacy of serves increased and the efficacy of side-out phases decreased. The game had a faster pace than with the standard rules but lowering the net reduced receivers' possibilities to send the ball to the setter. This resulted in reduced side-out efficacy and increased quality and efficacy of the defense. The imbalance between serve and reception occurred despite the jump serves not being allowed, and serves were limited to two serves per player to avoid more skillful players increasing the serve-reception balance.

In Experimental Tournament 2, in which there were a reduction in the net height and court size as well as a serve limitation, the game dynamics were different. There was a reduction in serve efficacy, although the quality of its execution increased. The serve speed was lower, probably due to the reduction of the court size. This involved increased quality and efficacy of the reception and increased efficacy of the side-out. The number of blocks and defenses increased. Players adopted better defense positions before the opponent's attack was executed, although the defense quality and efficacy decreased. There was greater continuity in the game (i.e., number of rallies per play). The increased continuity and the way the offense was built increased the number of jumps, number of hits taken by the players, and players' heart rate. There was a reduction in the time that players had to realize the reception, attack, and block due to the reduction in the distance of the court (court depth and width). The changes in Experimental Tournament 2 involved more balance between serve and reception which increased the efficacy of the side-out. The reduction in the court size meant that the distance between the setter and the attack destinations was smaller which allowed setters to increase the precision of their actions due to less force requirements. The change in the net height did not improve the attacker's efficacy, likely because the reduction of the net height was counter-balanced by the court size change (reduction of blockers' displacement, percentage of net zone covered by blockers, and percentage of court zone covered by defenders). However, there were more attacks and increased side-out efficacy during youth female volleyball matches. This net height reduction also involved more attacks being contacted by the blockers and defenders. The efficacy of the defense actions decreased, but, overall, there was an increase in the continuity of the game. The changes in the net height did not involve changes in the g-force in take-off and the landing of the attack. The limitations of the realization of jumps in the serve did not reduce the jumps done by players. These rule changes created game dynamics that were more similar to the progression found through the different developmental stages (García-de-Alcaraz et al., 2017; Echeverria et al., 2019). Side-out actions were done in situations that allowed players to have more successful experiences and happened with more frequency. Scaling the court size allowed players in this age group to better intercept the ball trajectories and interact with the ball. This increased the quality of the side-out, continuity, and participation in the game. The offense's success was achieved by having to overcome the block and defense that contacted the ball with more frequency. The reduction of the net height and court space involved players having to find different strategies to achieve points (e.g., increase attack speed, play against the block, etc.). The reason for the changes in Experimental Tournament 2 were the result of the combined impact of the limitations on the serve that were implemented (jump serve was not allowed and limit of two serves per player) and the changes in the net height and court size. Previous studies showed that a reduction of the court size increases the efficacy of the actions, except for the serve (Ronglan and Grydeland, 2006; Rocha et al., 2020a,b; Rodrigues et al., 2022). At the psychological level, none of the experimental tournaments involved changes regarding players' individual or collective self-efficacy, perceived enjoyment, or perceived satisfaction with regard to the Control Tournament. These findings could be due to the fact that this study assessed the immediate effect of the changes in the athletes' behaviors in a tournament. More studies are required to confirm this hypothesis.

The result of the current study must be interpreted with caution. The study only analyzed the immediate effect of the experimental rules in a specific group of players (three female regional teams) during two tournaments. Players did not practice using the experimental rules more than one day before the tournaments. This study does not allow us to establish the short-, medium-, or long-term impacts. This would require a research design that evaluates the impact of training and playing with these rules. More studies are needed with players of different levels (e.g., national and international levels) and sex. However, the results show that for this sample, scaling volleyball to players in U-14 may involve more players participation, quality of the execution, efficacy, and continuity. It is important to emphasize that this continuity did not just involve the ball passing over the net. To contribute to the better development of players, the game should involve and allow them to do successful varied actions. For that reason, it is important to study the interaction of the various rule changes and consider their combined impact. More studies are needed to establish which progressive evolution of rules is more appropriate for the different stages of development of youth volleyball players. These studies should analyze the effect of the different rule formats in each age group and their progression through the different stages of players development.

## Conclusion

The experimental rules that were tested had different impacts on the game dynamics and players' actions. Scaling the net and adapting the serve rules (Experimental Tournament 1) altered the balance between serve and reception toward the serve, which resulted in an increase in the efficacy of serves and a decrease in the efficacy of side-out phases. This imbalance reduced the attack and blocks as well as the efficacy of these actions. Scaling the net and court and adapting the serve rules (Experimental Tournament 2) altered the balance between serves and reception toward the reception which increased the efficacy of side-out phases. These changes involved an increase in reception efficacy, the occurrence of attacks and blocks, game continuity, and players' effort. For the U-12 teams that were studied, this combination of competition rules resulted in game dynamics that were more similar to those observed in later stages of player development (i.e., García-de-Alcaraz et al., 2017; Echeverria et al., 2019). None of the experimental conditions involved changes regarding self-efficacy, perceived enjoyment, or perceived satisfaction with regard to the Control Tournament.

The competition rules during developmental stages could have a critical role in the player's development. Therefore, future studies are necessary to establish the proper rules for each age group to facilitate appropriate player development. The development process should be analyzed as a whole considering the evolution of the competition rules in each age group, their synchronization, progression, and their relationship from a holistic perspective. In the past, due to most of the studies being done in physical education, the manipulation of constraints focused on achieving continuity (i.e., three contacts per team). Future research must focus on the quality of the movement done by players to acquire and practice proper ways of skill execution (mechanical performance and avoiding injuries). This should be the basis to ensure (a) the precision of the actions (sending the ball to the target) that will allow for continuity of the game and allow other players to carry out proper executions and (b) the speed and power of the players' actions that allow them to apply their physical capacities and do not limit future improvements. Developmental stages are the critical period to develop the technique and speed integrated into the game actions and context (adapted from Balyi and Hamilton, 2004; Lloyd and Oliver, 2012; Pichardo et al., 2018).

The intended practical application of this study is increased knowledge about the combined effect of the manipulation of the different sports constraints on volleyball game dynamics and the need to study this impact from several dimensions (technical, tactical, physical, psychological, etc.). The impact of scaling the net and court alters the balance between serve and reception, impacting the occurrence, quality, and efficacy of the posterior actions, as well as the game continuity and physical efforts. In this research, the progression of the game dynamics observed in older age groups was used as a reference. For that reason, the manipulation of net height and court size was completed with serve limitation rules to promote more balanced game dynamics with more quality, efficacy, continuity, and variability. Future experimental studies with an intervention training period should verify whether this proposed rule modification achieves that or not.

## Data availability statement

The raw data supporting the conclusions of this article will be made available by the authors, without undue reservation.

## Ethics statement

The studies involving humans were approved by University Ethics Committee of University of Murcia (ID 1944/2018). The studies were conducted in accordance with the local legislation and institutional requirements. Written informed consent for participation in this study was provided by the participants' legal guardians/next of kin. Written informed consent was obtained from the minor(s)' legal guardian/next of kin for the publication of any potentially identifiable images or data included in this article.

## Author contributions

JP: Conceptualization, Data curation, Investigation, Methodology, Validation, Writing—original draft. AU: Conceptualization, Supervision, Writing—review & editing. MM: Conceptualization, Supervision, Writing—review & editing. EO-T: Conceptualization, Formal analysis, Funding acquisition, Methodology, Project administration, Writing—original draft, Writing—review & editing.
