# Supplementary material for: Effect of changes in the net height, court size, and serve limitations on technical-tactical, physical, and psychological aspects of U-14 female volleyball matches
Source: Front Psychol. 2024 Jan 10;14:1341297. doi: 10.3389/fpsyg.2023.1341297 (PMC10806054; doi:10.3389/fpsyg.2023.1341297)
Supplement: Supplementary file 1 [file Table_1.DOCX]

Supplementary Material

APPENDIX 1. QUESTIONNAIRE OF SPECIFIC SELF-EFFICACY AND COLLECTIVE EFFECTIVENESS IN VOLLEYBALL

In the first column (individually), mark the degree to which you will feel that you were able to do the different game actions on a scale of 0 (not able) to 10 (completely able). In the second column (My teammates), mark the degree to which you will feel that they were able to do the different game actions on a scale of 0 (not able) to 10 (completely able).

|  | **INDIVIDUALLY** | **MY TEAMMATES** |
| --- | --- | --- |
| **GAME ACTIONS** | Not able Completely able | Not able Completely able |
| To what degree do you feel that you are able to serve? | 0 1 2 3 4 5 6 7 8 9 10 | 0 1 2 3 4 5 6 7 8 9 10 |
| To what degree do you feel that you are able of receive the opponent's serve with your teammates? | 0 1 2 3 4 5 6 7 8 9 10 | 0 1 2 3 4 5 6 7 8 9 10 |
| To what degree do you feel that you are able to set forward? | 0 1 2 3 4 5 6 7 8 9 10 | 0 1 2 3 4 5 6 7 8 9 10 |
| To what degree do you feel that you are able to set backward? | 0 1 2 3 4 5 6 7 8 9 10 | 0 1 2 3 4 5 6 7 8 9 10 |
| To what degree do you feel that you are able to realize a powerful attack? | 0 1 2 3 4 5 6 7 8 9 10 | 0 1 2 3 4 5 6 7 8 9 10 |
| To what degree do you feel that you are able to realize a controlled attack? | 0 1 2 3 4 5 6 7 8 9 10 | 0 1 2 3 4 5 6 7 8 9 10 |
| To what degree do you feel that you are able to attack a high ball that is separated from the net? | 0 1 2 3 4 5 6 7 8 9 10 | 0 1 2 3 4 5 6 7 8 9 10 |
| To what degree do you feel that you are able to organize the attack with your teammates? | 0 1 2 3 4 5 6 7 8 9 10 | 0 1 2 3 4 5 6 7 8 9 10 |
| To what degree do you feel that you are able to block with your teammates? | 0 1 2 3 4 5 6 7 8 9 10 | 0 1 2 3 4 5 6 7 8 9 10 |
| To what degree do you feel that you are able to defend with your teammates? | 0 1 2 3 4 5 6 7 8 9 10 | 0 1 2 3 4 5 6 7 8 9 10 |
| To what degree do you feel that you are able to defend the opponent's attack with your teammates? | 0 1 2 3 4 5 6 7 8 9 10 | 0 1 2 3 4 5 6 7 8 9 10 |
| To what degree do you feel that you are able to defend a powerful attack? | 0 1 2 3 4 5 6 7 8 9 10 | 0 1 2 3 4 5 6 7 8 9 10 |
| To what degree do you feel that you are able to defend a tip? | 0 1 2 3 4 5 6 7 8 9 10 | 0 1 2 3 4 5 6 7 8 9 10 |
| To what degree do you feel that you are able to organize the counter-attack with your teammates? | 0 1 2 3 4 5 6 7 8 9 10 | 0 1 2 3 4 5 6 7 8 9 10 |
